# Intensity-Modulated Proton Therapy for Nasopharynx Cancer: 2-year Outcomes from a Single Institution

**DOI:** 10.14338/IJPT-20-00057.1

**Published:** 2021-04-22

**Authors:** Vonetta M. Williams, Upendra Parvathaneni, George E. Laramore, Saif Aljabab, Tony P. Wong, Jay J. Liao

**Affiliations:** 1 Department of Radiation Oncology, University of Washington, Seattle, WA, USA; 2 Department of Radiation Oncology, Roswell Park Comprehensive Cancer Center, Buffalo, NY, USA; 3 Seattle Cancer Care Alliance Proton Therapy Center, Seattle, WA, USA

**Keywords:** proton therapy, nasopharyngeal cancer, head and neck cancer

## Abstract

**Purpose:**

Advances in radiotherapy have improved tumor control and reduced toxicity in the management of nasopharyngeal carcinoma (NPC). Local failure remains a problem for some patients with advanced primary tumors, and toxicities are significant given the large treatment volume and tumor proximity to critical structures, even with modern photon-based radiotherapy. Proton therapy has unique dosimetric advantages, and recent technological advances now allow delivery of intensity-modulated proton therapy (IMPT), which can potentially improve the therapeutic ratio in NPC. We report our 2-year clinical outcomes with IMPT for NPC.

**Materials and Methods:**

We retrospectively reviewed treatment records of patients with NPC treated with IMPT at our center. Demographics, dosimetry, tumor response, local regional control (LRC), distant metastasis, overall survival, and acute and late toxicity outcomes were reviewed. Analyses were performed with descriptive statistics and Kaplan-Meier method. Toxicity was graded per Common Terminology Criteria for Adverse Events (version 4.0).

**Results:**

Twenty-six patients were treated from 2015 to 2020. Median age was 48 years (range, 19–73 years), 62% (n = 16) had T3-T4 disease, 92% (n = 24) were node positive, 92% (n = 24) had stage III-IV disease, and 69% (n = 18) had positive results for Epstein-Barr virus. Dose-painted pencil-beam IMPT was used. Most patients (85%; 22 of 26) were treated with 70 Gy(RBE) in 33 fractions once daily; 4 (15%) underwent hyperfractionated accelerated treatment twice daily. All received concurrent cisplatin chemotherapy; 7 (27%) also received induction chemotherapy. All patients (100%) completed the planned radiotherapy, and no acute or late grade 4 or 5 toxicities were observed. At median follow-up of 25 months (range, 4-60), there were 2 local regional failures (8%) and 3 distant metastases (12%). The Kaplan-Meier 2-year LRC, freedom from distant metastasis, and overall survival were 92%, 87%, and 85% respectively.

**Conclusion:**

IMPT is feasible in locally advanced NPC with early outcomes demonstrating excellent LRC and favorable toxicity profile. Our data add to the growing body of evidence supporting the clinical use of IMPT for NPC.

## Introduction

Nasopharyngeal carcinoma (NPC) is a relatively rare cancer that accounts for < 1% of all cancers worldwide [1]. It is uncommon in North America, but the prevalence is significantly greater in certain regions of the world where it is endemic, including Southeast Asia, Northern Africa, and the Middle East. NPC has a distinct natural history and unique management considerations compared with more common mucosal squamous cell carcinomas of the head and neck.

NPC is sensitive to both chemotherapy and radiation. Primary radiotherapy (RT) has long been the mainstay of treatment for NPC. Numerous clinical trials in patients with locally advanced disease established that the addition of chemotherapy to radiation therapy improves local regional control (LRC) and survival, and chemoradiation is now the accepted standard of care in these patients [2, 3].

Treatment of NPC with RT involves targeting detectable gross disease as well as a large and complex volume of subclinical risk encompassing the nasopharynx, skull base neural pathways, and bilateral cervical and retropharyngeal lymph node regions. Many NPCs are challenging to treat because of their close proximity to, or invasion of, critical neural structures, such as the temporal lobe brain, brainstem, spinal cord, orbits, optic structures, and cochlea. Prior advances in RT with intensity-modulated radiation therapy (IMRT) improved the outcomes in NPC by facilitating superior coverage of treatment volumes and reducing doses to critical structures compared with older techniques. A number of studies, including several randomized trials, showed that the use of IMRT can improve local control and reduce toxicity, including xerostomia and neurologic toxicities [4, 5]. Long-term follow-up of IMRT series, both in North American [6–9] and in endemic populations [10, 11], have established this as a standard for NPC.

There is still significant room for improvement. Local failure remains a problem for many patients with locally advanced primary tumors. Acute toxicity of concurrent chemoradiation is significant, and this continues to be a very challenging treatment regimen requiring substantial supportive care. Fortunately, many patients are cured, but many long-term survivors are subject to significant late effects of treatment, including xerostomia, dysphagia, neurologic complications, vision or hearing loss, endocrine dysfunction, and cognitive effects [12].

Proton beam therapy (PT) has unique dosimetric properties related to the deposition of radiation at a Bragg peak at the end of the range of the particle with a lack of exit dose and, therefore, a sharp dose fall-off. This may further improve the therapeutic ratio by reducing the doses to healthy tissues and critical structures out of target volumes compared with photon-based approaches. In addition, PT technology itself has evolved over time. Most early experiences with PT were delivered using passive-scatter proton therapy (PS-PT), also referred to as 3-dimensional conformal PT. PS-PT is delivered using scattering foils, range modulation, custom compensators, and brass apertures. The use of PS-PT has been reported in the management of head and neck cancers as primary therapy and in the setting of reirradiation. However, PS-PT has several limitations, which have limited its use for treating head and neck tumors: (1) the need for fabrication of labor-intensive custom patient devices, (2) the lack of conformality of treatment volumes at the proximal target, and (3) the difficulty of treating comprehensive pharynx and bilateral neck target volumes with protons alone, requiring either complex matching techniques or mixed-beam approaches of combined protons and photons. Several early experiences with PS-PT in NPC have been reported with encouraging clinical outcomes, primarily as a mixed-beam approach combined with photons [13–15].

Technological advances have made delivery of intensity-modulated proton therapy (IMPT) feasible, also referred to as pencil beam therapy, which uses magnetic steering of a pencil beam to create layers of spots covering the tumor volume without compensators or aperture devices. This allows superior conformality compared with PS-PT, reduction in integral dose, and the ability to dose-paint target volumes. Therefore, IMPT is well suited to the management of NPC given the need to treat an anatomically complex target volume in close proximity to numerous critical structures.

Dosimetric analyses have shown that IMPT can provide superior sparing of numerous critical structures compared to IMRT [16–19]. Lewis et al [16] reported the earliest experience of IMPT for NPC in 10 patients with encouraging results, but there is still relatively limited clinical data reporting on outcomes using IMPT for NPC. We evaluated our initial experience with IMPT in the definitive management of NPC at our institution. We report our 2-year clinical results, including oncologic outcomes, patterns of failure, and toxicity measures.

## Materials and Methods

### Study Design

This was a single-institution retrospective review of patients with NPC treated at our center, the University of Washington, Seattle Cancer Care Alliance Proton Therapy Center, from 2015 to 2020. The study period was selected to begin in 2015, when IMPT became available at our center. The study was reviewed and approved by our institutional review board, and all patients were enrolled on a clinical registry study. Eligibility criteria included patients who had (1) a pathologically confirmed diagnosis of NPC, (2) treatment with curative intent with primary RT using IMPT, (3) no prior head and neck RT, and (4) a minimum follow-up of 3 months. Clinical endpoints included tumor response (primary and neck), LRC, distant metastasis (DM)-free survival, and overall survival (OS). We reviewed dosimetric parameters and acute and late toxicity outcomes.

### Patient Evaluation

Initial consultation and examination included flexible fiberoptic nasal endoscopy and was followed by a multidisciplinary tumor board discussion. Staging evaluation included diagnostic computed tomography (CT) and magnetic resonance imaging (MRI) scans, and systemic staging with CT of the chest, abdomen, and pelvis or a fluorodeoxyglucose positron emission tomography (PET)/CT scan. All patients underwent pretreatment dental clearance. Dental metal amalgam fillings and crowns potentially in the beam path were replaced when needed by tissue-density composite material to minimize the risk of beam path dose uncertainties [20]. Patients were evaluated for prophylactic percutaneous endoscopy gastrostomy (PEG) placement. In general, our institutional practice has been to encourage prophylactic PEG placement pretreatment or early in treatment in most patients. In addition, PEG was strongly advised in patients with significant pretreatment weight loss or those felt to be at high risk for excessive weight loss during chemoradiation. In those patients who declined upfront PEG placement, a reactive PEG placement during treatment was considered if patients were unable to tolerate oral intake and/or lost 10% body weight during treatment.

### Radiotherapy Planning and Treatment

**Figure 1.**
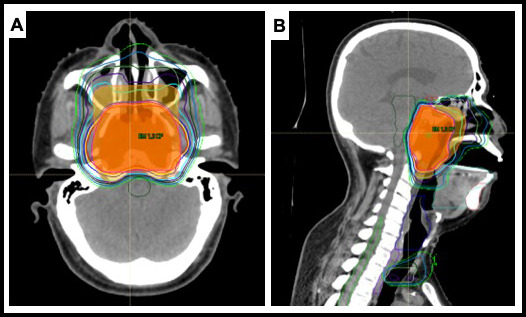
Pencil-beam scanning intensity-modulated proton therapy (IMPT) plan, 2-fields, right anterior oblique and left anterior oblique; planning target volume 1 (PTV1), shown in shaded red, was 70 Gy(RBE) in 33 fractions, 2.12 Gy(RBE) per fraction. Low- and high-risk subclinical volumes received 54 Gy(RBE) (not shown) and 60 Gy(RBE) (shaded orange), respectively. (A) axial view. (B) Sagittal view.

Treatment simulation for proton therapy was performed with a noncontrast and contrast helical CT scan (1.25-2.5-mm slices) with patients immobilized in a head, neck, and shoulder thermoplastic mask, BoS table (Qfix, Avondale, Pennsylvania), custom MoldCare cushion (Qfix), and custom oral stent for jaw and tongue immobilization, as previously described [21]. Image registration and fusion with diagnostic MRI and PET/CT scans were performed per standard practice. Target volumes and organ at risk volumes were delineated by the treating radiation oncologist on each axial CT slice according to standard contouring guidelines. The prescribed IMPT radiation doses were specified using a relative biologic effectiveness (RBE) value of 1.1. Most patients (85%; 22 of 26) were treated to 70 Gy(RBE) in 2.12 Gy(RBE) in 33 fractions, delivered once daily, 5 days a week, to the planning target volume encompassing gross disease (PTV1). Dose-painted plans were generated, with areas deemed at higher risk of subclinical disease receiving 59.4 to 63 Gy(RBE) and areas deemed at lower risk of subclinical disease receiving 54 to 57 Gy(RBE). Typically, 3- to 5-mm margins were used for PTV expansions. An illustrative IMPT plan for a patient with NPC is shown in **Figure 1**.

The IMPT planning was performed using RayStation (RaySearch Laboratories, Stockholm, Sweden). All patients were treated to the nasopharynx and bilateral neck with a 2- to 5-field pencil-beam scanning IMPT plan. The standard beam arrangement consisted of a 3-field approach (posterior-anterior, left anterior oblique, and right anterior oblique), and an anterior fourth beam was added in some cases. Initially, we used single-field optimization with split-target junction matching but subsequently transitioned to multifield optimization as our standard in 2018. Dose calculation was initially performed with a pencil-beam algorithm, but we transitioned to a Monte-Carlo algorithm in 2017. Details of our approach have been previously published [22]. Treatment-plan robustness analysis was performed with a combination of PTV optimization objectives and clinical target volume–based robust optimization. The nominal plan was recalculated for 8 different robustness evaluation scenarios: ± 3% range uncertainties and ± 3-mm setup uncertainty in 6 translational setup directions (right-left, anterior-posterior, and superior-inferior). These evaluation scenarios meet our criteria of covering ≤ 95% of the clinical target volume with 95% of the prescribed dose. Patient plan-specific quality-assurance testing was performed by the medical physics team.

During treatment, daily orthogonal 2-dimensional kV images were obtained with digitally reconstructed radiographs for patient alignment. Image-guided radiation therapy was performed by acquiring orthogonal 2-dimensional kV images, which were registered with 2 digitally reconstructed radiographs rendered from the planning CT. The image-guided radiation therapy system (Verisuite, Medcom, Germany) calculated the setup errors and provided 6° of freedom correction vectors with submillimeter accuracy. Translational setup errors were sent to the treatment control system to correct the treatment setup with a residual tolerance of 2 mm between the planned and treatment setup. Quality-assurance CT scans were performed per physician discretion during the treatment course to verify the consistency of the setup and to assess any interval anatomic changes. Typically, this was performed at least once midcourse. Adaptive replanning was performed at the discretion of the primary radiation oncologist when indicated (ie, significant patient weight loss or interval tumor regression from treatment response).

### Chemotherapy

All patients (100%; 26 of 26) received concurrent cisplatin (CDDP) chemotherapy administered either once every 21 days (3 cycles) or low dose weekly, with or without induction chemotherapy. Induction chemotherapy was considered in patients with very advanced local and/or regional disease or who were very symptomatic or had rapidly progressive disease at presentation, in order to expedite initiation of tumoricidal therapy and to allow time for disease response and complex IMPT planning.

### Evaluation and Data Collection

Patients were routinely assessed weekly during RT. Toxicity endpoints were assessed and documented by the treating physician. Posttreatment evaluations were performed per our standard institutional practice: every 3 months for the first year, every 4 months for the second year, and then, every 6 to 12 months for 3 to 5 years. Restaging evaluations consisted of physical exam, office endoscopy, and imaging with CT and/or MRI scan. If clinically indicated, a PET/CT scan was obtained.

Acute toxicity was defined from the start of radiotherapy to 3 months after treatment, and late toxicity was defined from 3 months after IMPT. Data collection was performed retrospectively via the electronic medical records. Toxicities were graded according to Common Terminology Criteria for Adverse Events (CTCAE), version 4.0 [23]. Primary tumor complete response (CR) was defined as no evidence of tumor based on a clinical exam and follow-up imaging, partial response (PR) was defined as > 50% tumor reduction, and no response (NR) was defined as no reduction in tumor size or any evidence of progression. Neck nodal CR was defined on imaging as complete nodal resolution or reduction to < 1.5 cm in maximal dimension without suspicious morphology (ie, central necrosis); PR was defined as > 1.5-cm residual lymph node, and NR was defined as no reduction in nodal size or any evidence of progression.

### Statistical Analysis

Descriptive statistics were used to assess the baseline characteristics of the patients and their tumor response. Survival times were calculated from the IMPT end date to the occurrence of the first event. Follow-up was calculated from the IMPT end date. Primary site local recurrence and/or neck recurrence were considered events for LRC analysis, and DM was considered an event for freedom-from-DM analysis. Death from any cause was considered an event for OS. Survival rates were estimated with the Kaplan-Meier (KM) method, and patients were censored after a recorded event or last follow-up visit. Statistical analyses were performed with Prism software (version 8.0; GraphPad Software, La Jolla, California).

## Results

### Patient, Tumor and Treatment Characteristics

Twenty-six patients were treated from 2015 to 2020. Patient and tumor characteristics are shown in **Table 1**. Most patients (89%; n = 23) had World Health Organization type II or III disease. All but 2 patients (92%; n = 24) had Epstein-Barr virus (EBV) testing by EBV-encoded small RNA fluorescence in situ hybridization, and 69% (n = 18) had positive EBV results. Assessment of p16 status was not consistently performed. One patient (6%) was p16 positive, 16 patients (94%) were p16 negative, and 9 (35%) had unknown p16 status. Most patients had advanced primary tumors with 15 (58%) having T4 tumors and 14 (54%) having advanced nodal disease N2/3. Almost all patients (92%; n = 24) had node-positive disease and stage III to IVA disease as defined by the AJCC 8th edition [24].

**Table 1.** Patient and tumor characteristics (N = 26).

**Characteristic**	**Value**
Median age, y, median (range)	48 (19-73)
Gender, No. (%)	
Male	18 (69)
Female	8 (31)
WHO classification, No. (%)	
I	3 (12)
II	2 (8)
III	21 (81)
EBV status, No. (%)	
Positive	18 (69)
Negative	6 (23)
Unknown	2 (8)
T category, No. (%)	
1	5 (19)
2	5 (19)
3	1 (4)
4	15 (58)
N category, No. (%)	
0	2 (8)
1	10 (38)
2	11 (42)
3	3 (12)
Stage	
II	2 (8)
III	7 (27)
IVA	17 (65)
Concurrent chemotherapy, No. (%)	
CDDP every 3 wk	22 (85)
Weekly CDDP	4 (15)
Induction chemotherapy	7 (27)

Abbreviations: WHO, World Health Organization; EBV, Epstein-Barr virus; CDDP, cisplatin

All patients (100%) completed the planned RT course with no unplanned treatment breaks. Twenty-two patients (85%) were treated to 70 Gy(RBE) in 33 fractions once daily. Four patients (15%), who had primary tumors with extensive skull base invasion, were treated with a hyperfractionated accelerated course delivered twice daily to 66 to 72 Gy(RBE) in 55 to 60 fractions of 1.2 Gy(RBE) per fraction. All patients (100%) received concurrent CDDP chemotherapy delivered every 3 weeks (85%; n =22 ) or low dose weekly (15%; n = 4). Of the patients who received high dose CDDP delivered every 3 weeks (n = 22; 85%), all were able to complete 2 (5 of 22; 23%) or 3 cycles (17 of 22; 77%). Seven patients (27%; 7 of 26) also received induction chemotherapy for 1 to 2 cycles before concurrent chemoradiation. Regimens included docetaxel, cisplatin, and fluorouracil (14%; 1 of 7); gemcitabine/carboplatin (43%; 3 of 7), docetaxel/CDDP (14%; 1 of 7), and gemcitabine/CDDP (29%; 2 of 7). No patients received adjuvant chemotherapy.

### Dosimetric Analysis

The dose-calculation algorithm was pencil-beam in 15 patients (58%) and Monte-Carlo in 11 patients (42%). The PTV coverage was excellent with median D95 (dose covering 95% of the volume) for the high-dose PTV1 = 100% of prescription, and all but one patient (25 of 26; 95%) had a D95% > 95% (range, 94%-100.5%). Dosimetric parameters for organs at risk with median and interquartile ranges are shown in **Table 2**. As expected, the degree of achievable salivary gland sparing was highly dependent on both primary and nodal tumor extent as well as patient anatomy. Median of the mean parotid gland dose was 31 Gy, and 17 patients (65%) had at least 1 parotid gland with a mean dose ≤ 26 Gy. Median of the mean parotid gland dose for uninvolved necks was 23 Gy and for involved necks was 33 Gy. Median of the mean submandibular gland dose was 36 Gy, and 20 patients (77%) had at least 1 submandibular gland with mean dose ≤ 39 Gy. Median of the mean cochlea dose was 31 Gy. Median of the mean larynx dose (which included the inferior pharyngeal constrictor muscle) was 24 Gy. An oral-cavity avoidance structure used for dose optimization was defined as uninvolved mucosa of the oral tongue, floor of mouth, and buccal mucosa. Median of the mean uninvolved oral cavity was 14 Gy. Doses to critical neural structures were also highly dependent on primary tumor extent and perineural spread. Median of the maximum dose to brainstem was 54 Gy; spinal cord, 37 Gy; optic nerves, 35 Gy; and optic chiasm, 42 Gy.

**Table 2.** Dosimetric parameters and organs at risk.

**Structure**	**Median cGy (RBE)**	**Interquartile Range cGy (RBE)**
Brainstem (maximum)	5480	4782–5957
Spinal cord (maximum)	3704	2400–4066
Optic chiasm (maximum)	4172	2725–5021
Optic nerves (maximum)	3473	2697–5043
Cochlea (maximum)	3085	2494–4276
Oral cavity (mean)	1417	1135–2208
Larynx/inferior constrictors (mean)	2468	1963–3057
Parotid, involved neck (mean)	3357	2840–3995
Parotid, uninvolved neck (mean)	2322	1970–2536
Submandibular gland (mean)	3619	3280–4021

### Tumor Response and Survival Analysis

Primary tumor response was assessed as CR in 24 patients (92%), PR in 1 patient (4%), and NR in 1 patient (4%). The patient with PR had a T4 primary with > 50% reduction and residual soft tissue and clival bone erosion, which may be treatment effect. This patient is doing well clinically at 20 months posttreatment without disease progression or recurrence. One local failure (4%) was observed, which was the patient with NR who had a T4 primary, World Health Organization Type I . This patient progressed at the skull base early after completion of treatment superior to the high-dose PTV volume and died of disease.

Neck nodal tumor response was assessed as CR in 24 patients (92%) and PR in 2 patients (8%). One patient with PR has a persistent but stable node and also developed distant metastasis, is currently alive receiving systemic therapy. The other patient with PR had massive neck lymphadenopathy > 10 cm at diagnosis and, notably, also had synchronous advanced lymphoma. This latter patient achieved a marked response > 50% but developed distant metastasis as well as neck recurrence with extensive dermal and subcutaneous metastasis both within and out of the field and died of disease.

Three patients (12%) developed distant metastatic disease at 1, 7, and 14 months after treatment, respectively. All (100%; 3 of 3) had multiple sites of distant metastasis, which included lung, liver, bone, and nodal involvement. Two of the aforementioned patients (67%) died of disease, and 1 patient (33%) died of unrelated causes, related to complications of preexisting chronic lung disease.

**Figure 2.**
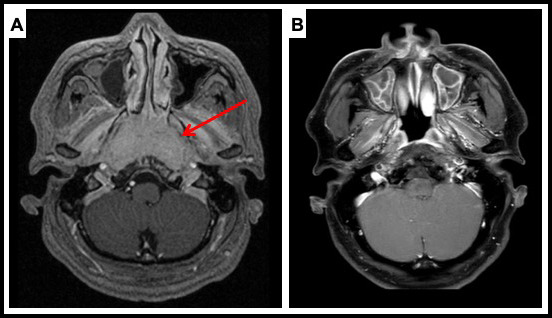
Patient case: 47-year-old male with locally advanced, undifferentiated, nonkeratinizing nasopharyngeal carcinoma (NPC), cT4N2M0 Epstein-Barr virus (EBV)-positive tumor treated with chemoradiation with intensity-modulated proton therapy (IMPT). The IMPT plan is shown in Figure 1. (A) Pretreatment magnetic resonance imaging (MRI; T1 postcontrast magnetization-prepared rapid acquisition gradient echo [MPRAGE] sequence) showing a large nasopharyngeal mass with central skull base invasion. He also had bilateral retropharyngeal and cervical lymphadenopathy. (B) Posttreatment MRI (T1 postcontrast fat sat [saturation] sequence) showing resolution of his extensive nasopharyngeal primary, with some benign posttreatment sinonasal mucosal changes. He had a complete remission (CR) and remains without evidence of disease now 3 years after treatment.

An illustrative patient treated with IMPT is shown in **Figure 2**, with pre-treatment and post-treatment imaging. This patient had an extensive T4 primary tumor with central skull base invasion and had presented with diplopia from an abducens palsy. With IMPT, mean doses to his bilateral cochlea were able to be kept low at 32 Gy(RBE), despite tumor invasion involving the bilateral petrous apex in close proximity. He achieved a CR and remains without evidence of disease at his 3-year follow-up. His hearing remains intact, as does his vision, with resolution of his abducens palsy.

**Figure 3.**
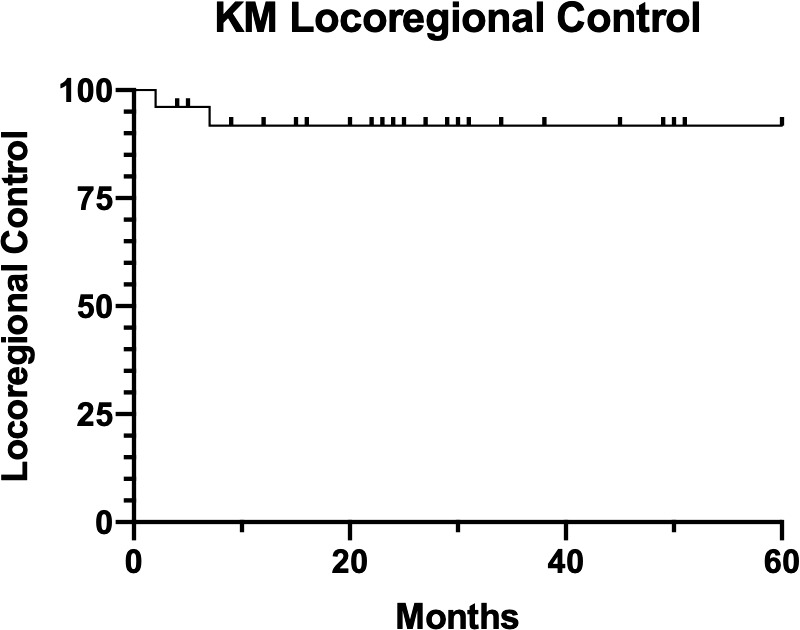
Kaplan-Meier (KM) local regional control.

**Figure 4.**
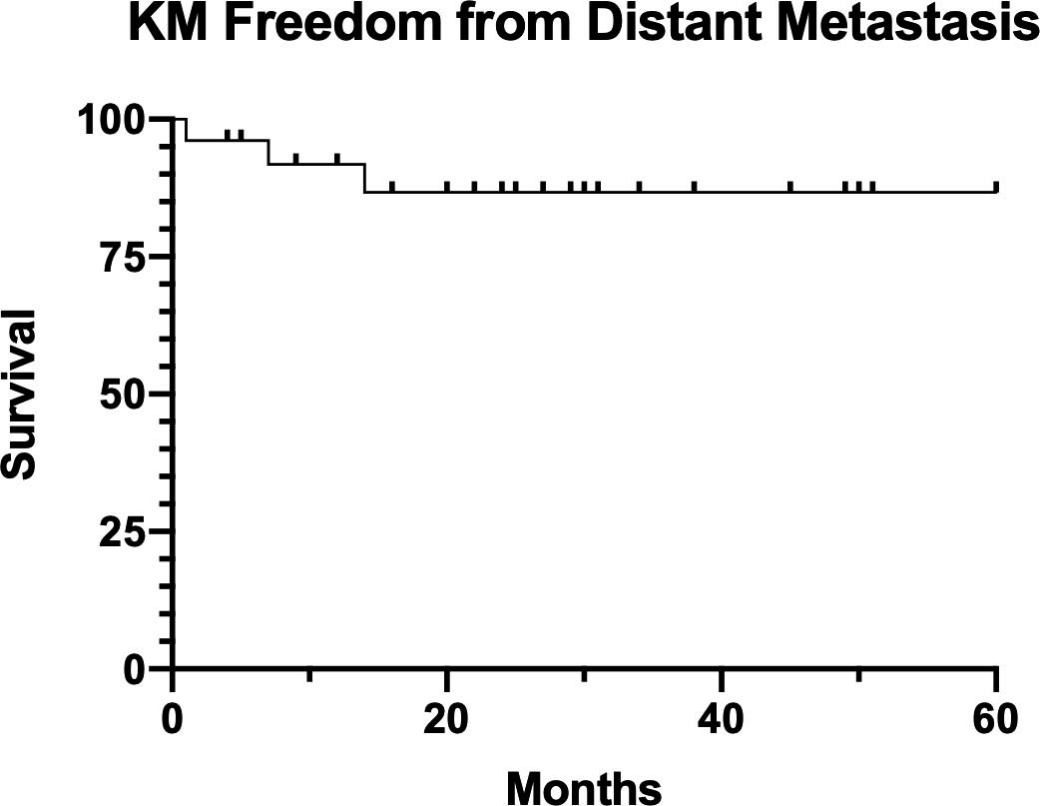
Kaplan-Meier (KM) freedom from distant metastasis.

**Figure 5.**
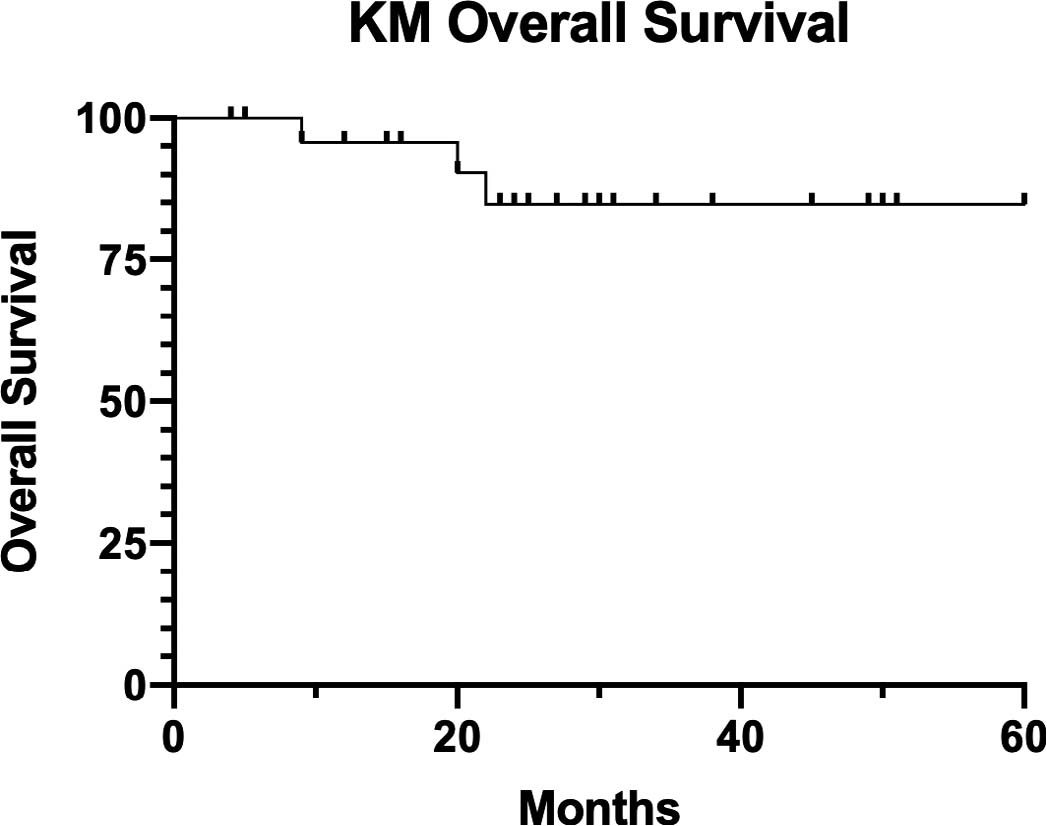
Kaplan-Meier (KM) overall survival.

Median follow-up for our patient cohort is 25 months (range, 4-60 months). The KM 2-year LRC rate was 92%, shown in **Figure 3**.The KM 2-year freedom from DM was 87%, shown in **Figure 4**. KM 2-year OS was 85%, shown in **Figure 5**.

### Toxicity

Treatment was completed in all patients without significant treatment interruptions during IMPT. Three patients (12%) had emergency room visits: one (33%) for a vasovagal event, one for hydration (33%), and one (33%) for chest pain for which cardiac etiology was ruled out and resolved. One patient (4%) had a hospital admission for 2 days for management of an acute ischemic stroke, which occurred during neoadjuvant chemotherapy before the start of IMPT. This patient recovered and completed IMPT.

Acute and late toxicities are shown in **Table 3**. No acute or late grade 4 or 5 toxicities were observed. Acute toxicities included 11 patients (42%) with grade 3 dermatitis, and 12 patients (46%) with grade 3 mucositis (CTCAE version 4.0 functional assessment). We also reviewed Radiation Therapy Oncology Group–based mucositis grading, which included 9 patients (35%) with grade 2 and 17 patients (65%) with grade 3 mucositis. Of note, although confluent mucositis was observed in a number of patients, this typically demonstrated a pattern of very focal and confined mucositis involving only the posterior soft palate, with sparing of the remainder of the oral cavity. In many patients, this did not result in intolerance to oral intake.

**Table 3.** Acute and late toxicity (Common Terminology Criteria for Adverse Events, version 4.0).

**Toxicity^a^**	**Grade, No. (%)**			
	**0**	**1**	**2**	**3**
Acute				
Dermatitis	0 (0)	6 (23)	9 (35)	11 (42)
Mucositis	0 (0)	5 (19)	9 (35)	12 (46)
Weight loss	9 (35)	12 (46)	5 (19)	0 (0)
Xerostomia	3 (12)	5 (19)	15 (58)	3 (12)
Dysgeusia	0 (0)	7 (27)	17 (65)	2 (8)
Chronic				
Dysphagia	21 (81)	4 (15)	1 (4)	0 (0)
Xerostomia	7 (27)	17 (65)	2 (8)	0 (0)
Dysgeusia	14 (54)	12 (46)	0 (0)	0 (0)
Hearing changes	18 (69)	7 (27)	1 (4)	0 (0)
Visual changes	23 (88)	3 (12)	0 (0)	0 (0)

a No grade 4 or 5 events were recorded for either acute or late toxicities.

Weight loss occurred in 17 patients (65%), including grade 1 (> 5%) in 12 (71%) and grade 2 (> 10%) in 5 patients (29%). Use of PEG included prophylactic placement in 11 patients (42%), reactive placement in 5 patients (19%), and none in 10 patients (38%). There are no patients with long-term PEG dependence. All patients have had their PEG tubes removed, except one patient (4%) who is 4 months posttreatment (anticipating upcoming removal) and another patient (4%) who died before their PEG was removed. The median time from PEG placement to removal for all patients was 126 days and, for patients with prophylactic placement, 99 days.

In terms of long-term toxicities, no grade 3 or higher toxicities have been observed. There are 21 patients (88%) with 12-month posttreatment assessments available. There was excellent recovery of taste, saliva, and swallowing, with a most patients reporting only grade 0 to 1 xerostomia, dysgeusia, and dysphagia. Reduced visual acuity was reported in 3 patients (12%), all of which were grade 1 (mild, intervention not indicated), and hearing loss was reported in 8 patients (31%), including grade 1 (mild subjective change in hearing) in 7 (88%) and grade 2 (hearing aid or intervention not indicated) in one patient (12%). No patients have developed radiation brain necrosis (either radiographic or clinical), osteoradionecrosis ,or late mucosal ulceration. One patient (4%) underwent functional endoscopic sinus surgery for chronic sinusitis after treatment, which has now resolved.

## Discussion

The role of proton therapy in the treatment of head and neck cancer continues to be defined by the ongoing work of numerous centers. The dosimetric advantages of proton therapy are well-suited to head and neck radiotherapy because LRC is critical, and tumor control is often closely related to radiation dose delivered to the tumor. Moreover, many head and neck tumors, such as NPC, lie in close relationship to numerous critical structures, including neural structures, and others that are important to long-term quality of life, including salivary glands, swallowing structures, oral cavity, and larynx. Recent advances in IMPT have improved conformal RT compared with older PS-PT techniques and allow treatment of more-complex tumor volumes as well as dose painting. In addition, comprehensive treatment of neck target volumes can be delivered with IMPT alone, without combined approaches with photons to address the neck or complex match techniques that were necessary with PS-PT techniques [13, 14, 25].

There is a growing body of evidence evaluating outcomes of proton therapy in several head and neck tumor sites [26]. Sinonasal cancers represent an anatomic site that is well suited for application of proton therapy. A large meta-analysis of 41 observational studies compared charged-particle therapy with photon therapy in paranasal sinus and nasal cavity cancers and found that proton therapy was associated with significantly greater 5-year disease-free survival (DFS) and LRC at longest follow-up compared with IMRT [27]. Several centers have reported favorable outcomes with proton therapy, primarily with the PS-PT technique, for sinonasal cancers with encouraging rates of LRC and favorable toxicity profile [28–30]. There are fewer data available for single-modality IMPT for head and neck cancer, but several centers, including ours, have reported encouraging early outcomes of IMPT in oropharyngeal cancer [31, 32]. These series demonstrate high rates of LRC and favorable side-effect profile at 2 years.

In NPC, early outcomes using PS-PT have been reported, primarily with a mixed-beam approach of photon therapy combined with PS-PT. Investigators from Massachusetts General Hospital (Boston) reported, in abstract form, a series of 17 patients with T4 NPC treated with a mixed-beam approach of photons and PS-PT [13]. At median follow-up of 43 months, there was 1 local and 2 distant failures with 3-year LRC of 92%, DFS of 75%, and OS of 74%. Late toxicities included radiographic changes of the temporal lobes (n = 5), mandibular osteoradionecrosis (n = 1), and endocrine dysfunction (n = 2). Chan et al [14] also reported, in abstract form, early outcomes of a phase II trial in 23 patients with NPC treated with a mixed-beam approach using PS-PT (nasopharynx/upper neck) and photons (low neck). At 28 months of follow-up, the 2-year LRC was 100%, DFS of 90% and OS of 100%. Beddock et al [15] reported a series of 17 NPC patients treated with a mixed-beam approach of photons combined with PS-PT boost. The 5-year local-recurrence–free survival was 86%, and toxicities included temporal lobe necrosis in 6 patients (1 symptomatic) and one patient with nasopharyngeal ulceration and necrosis.

There are still limited data reported on IMPT for NPC. Several reports have evaluated comparative dosimetric advantages of IMPT versus IMRT. Those studies demonstrate that IMPT can provide superior sparing of numerous critical structures and maintain tumor coverage [17–19]. Lewis et al [16] compared IMPT with IMRT plans in 9 patients with NPC and showed that IMPT reduced mean doses to 13 organs at risk, notably oral cavity, brain, spinal cord, cochlea, larynx, and esophagus. Widesott et al [18] reported a dosimetric comparison of IMPT versus helical tomotherapy in 6 patients and found that IMPT reduced mean doses to the parotid glands and the esophagus.

Several groups [33, 34] have reported early outcomes of a mixed-beam approach of photons combined with IMPT for NPC. Alterio et al [33] reported a series of 27 patients with NPC treated with chemoradiation using a mixed-beam approach of IMRT (54-60 Gy) followed by an IMPT boost to gross disease (total 70-74 Gy[RBE]). At median follow-up of 25 months, LC was 96%. Park et al [34] reported a series of 35 patients treated with a mixed-beam approach of IMRT (18 fractions) followed by an IMPT boost (final 12 fractions). Compared with a cohort of patients treated with IMRT alone, the mixed-beam approach was associated with a reduction in grade 2 or greater mucositis and grade 2 or greater analgesic use.

Few series to date have reported on clinical outcomes of single-modality IMPT used as the primary modality for treatment of NPC. Investigators from MD Anderson Cancer Center (Houston, Texas) reported a series of 10 patients with NPC treated with IMPT [16]. At median follow-up of 24.5 months, no local failures were observed yet, with 2-year LRC of 100% and 2-year OS of 88.9%. There were no grade 4 or 5 acute toxicities and no grade 3 or greater late toxicities. Jiri et al [35] recently reported a series of 40 patients with NPC treated with IMPT and 2-year LC was 84%. To our knowledge, our present experience represents the largest US single-institution report of IMPT for NPC. Our clinical outcomes at 2 years compare favorably with the aforementioned studies. At median follow-up of 25 months, the KM 2-year LRC was 92% with only 2 (8%) local regional failures observed. The KM 2-year freedom from DM was 87%, and the KM 2-year OS was 85%. No acute grade 4 or greater toxicities were seen, and there were no late grade 3 or greater toxicities.

Concurrent chemoradiotherapy for NPC continues to be associated with substantial toxicity, even with modern radiotherapy approaches. The significant long-term quality-of-life (QOL) effects of radiotherapy and chemotherapy in long-term survivors of NPC treated with chemoradiation have been well recognized. McDowell et al [12] recently reported a comprehensive review. They highlight the extensive late-treatment toxicities that affect patient QOL, including neurotoxicity, such as cranial neuropathies, neurocognitive effects, hearing loss, endocrine dysfunction (thyroid and pituitary), fatigue, nutritional and dental problems, xerostomia, and dysphagia. There remain many unmet needs in terms of toxicity reduction in this population.

Proton therapy may further improve the therapeutic ratio to improve tolerance of patients undergoing chemoradiation. A large retrospective comparative effectiveness analysis compared proton therapy versus photon therapy in 1483 patients with locally advanced solid tumors (including head and neck, lung, and gastrointestinal tract cancers) who underwent concurrent chemoradiation [36]. Proton therapy was associated with a significantly lower relative risk of 90-day adverse events of grade ≥ 2 or ≥ 3, as well as lower risk of decline in performance status, while maintaining similar DFS and OS.

Early toxicity outcomes for IMPT in NPC, thus far, appear encouraging. Holliday et al [17] reported a case-match control study of patients with NPC treated with IMPT (n = 10) compared with IMRT (n = 20) and found that IMPT reduced the rate of gastrostomy tube insertion, which was strongly associated with reduction in oral cavity mean dose with IMPT. In our series, a gastrostomy tube was placed in 16 of 26 patients (62%), but most of these were prophylactic placement pretreatment reflecting our institutional preference of prophylactic placement in patients undergoing CDDP-based chemoradiation. It has been our observation that overall tolerance to chemoradiation in our patients with NPC who are treated with protons is improved compared with those treated with IMRT, so we have more recently shifted away from prophylactic placement in many patients. In our cohort, there are no patients with long-term PEG dependence.

In the pediatric patient population, NPC is very rare; however, concerns of late toxicities are even more pronounced in this cohort than in adults. Investigators from the University of Florida (Gainesville) reported a series of 17 pediatric and adolescent patients with NPC who were treated with induction chemotherapy, followed by modestly dose-reduced proton therapy (median dose of 61.2 Gy; 11 patients had mixed-beam approach with photons and PS-PT) with concurrent chemotherapy [37]. At median follow-up of 3 years, LRC, PFS, and OS were 100%. With longer follow-up, there is hope that proton therapy may help reduce toxicity in the brain and skull base region without compromising disease control in this patient population. These considerations are applicable to many adult patients with NPC who are diagnosed and treated at a relatively young age. The median age in our series was relatively young at 48 years, and 23% (6 patients) were younger than 40 years.

The current study does have limitations given its retrospective nature, small patient cohort, and relatively short follow-up. Direct comparison with the conventional IMRT approach is difficult to extrapolate given the small sample set and the heterogeneity of NPC. However, with the relatively low incidence of NPC in the United States, it is likely impractical to conduct a randomized trial of IMRT versus IMPT. It is possible that such a trial design could be led by investigators in areas in which NPC is endemic. It is important to analyze and report the outcomes of available single-institutional experiences, given the relatively smaller patient numbers seen in the United States.

Given the sharp dose gradients associated with IMPT, careful planning and consideration of plan robustness is essential to avoid risk of marginal recurrence. Reassuringly, LRC appears extremely high, and marginal recurrences are rare in our experience as well as in the several series that have been reported [16, 35]. However, these results are early, and it has been recognized that recurrences from NPC may be seen from 3 to 5 years after treatment, so careful tracking of long-term outcomes will be important [7]. Late toxicities will also be important to track. The aforementioned early experiences of mixed-beam photon combined with PS-PT did report a few patients who developed temporal lobe necrosis, osteoradionecrosis, and mucosal necrosis after treatment [13, 15]. With the improved conformal mapping of IMPT and the reduction in the integral dose, we anticipate that the risk of these complications will be reduced. In the present series, we have not observed any brain necrosis (either symptomatic or radiographic) or high-grade late vision or hearing complications, but we will continue to closely track this cohort.

Despite the non-randomized nature of this study, this is one of the few reports to date reporting clinical outcomes of IMPT for NPC. Early outcomes are very encouraging, both in terms of disease control as well as toxicity. As a result, IMPT has largely been adopted at our center as the preferred platform for RT delivery in most patients with NPC. Future work may involve comparing prospective patient-reported QOL outcomes among patients treated with IMPT versus IMRT. Chemoradiation trials that explore novel and targeted systemic therapy and immunotherapy approaches are necessary because distant metastasis remains a major barrier to improving long-term survival outcomes with NPC.

## Conclusion

Our results show that IMPT is feasible in locally advanced NPC with early clinical outcomes demonstrating excellent LRC and favorable toxicity profile. This is consistent with early results from other single institutions. Longer follow-up will be needed to continue to track long-term disease control outcomes. In addition, it will be important to evaluate whether late toxicities are reduced and whether that translates to improvements in QOL. We plan to continue to follow our cohort and report long-term outcomes. Prospective studies with the use of IMPT for NPC are warranted.
